# Fractionation Coupled to Molecular Networking: Towards Identification of Anthelmintic Molecules in *Terminalia leiocarpa* (DC.) Baill

**DOI:** 10.3390/molecules28010076

**Published:** 2022-12-22

**Authors:** Esaïe Tchetan, Sergio Ortiz, Pascal Abiodoun Olounladé, Kristelle Hughes, Patrick Laurent, Erick Virgile Bertrand Azando, Sylvie Mawule Hounzangbe-Adote, Fernand Ahokanou Gbaguidi, Joëlle Quetin-Leclercq

**Affiliations:** 1 Laboratoire d’Ethnopharmacologie et de Santé Animale, Faculté des Sciences Agronomiques, Université d’Abomey-Calavi, Cotonou 01 BP 526, Benin; 2 Laboratoire de Biotechnologie et d’Amélioration Animale, Faculté des Sciences Agronomiques, Institut des Sciences Biomédicales Appliquées (ISBA), Université d’Abomey-Calavi, Cotonou 01 BP 526, Benin; 3 Laboratoire de Chimie Organique et Chimie Pharmaceutique, UFR Pharmacie, Faculté des Sciences de la Santé, Université d’Abomey-Calavi, Cotonou 01 BP 188, Benin; 4 Pharmacognosy Research Group, Louvain Drug Research Institute, Université Catholique de Louvain (UCLouvain), Avenue E. Mounier, 72, B1.72.03, B-1200 Brussels, Belgium; 5 UMR CNRS Laboratoire d’Innovation Thérapeutique (LIT) 7200, Faculté de Pharmacie, Université de Strasbourg, 74 Rte du Rhin, 67400 Illkirch-Graffenstaden, France; 6 Unité de Recherche en Zootechnie et Système d’Elevage (EGESE), Laboratoire des Sciences Animale et Halieutique (LaSAH), Ecole de Gestion et d’Exploitation des Sytèmes d’Elevage (EGESE), Université Nationale d’Agriculture (UNA), Porto-Novo 01 BP 55, Benin; 7 Laboratory of Neurophysiology, ULB Neuroscience Institute (UNI), Université Libre de Bruxelles (ULB), 808 route de Lennik, CP601, 1070 Brussels, Belgium; 8 Laboratoire d’Écologie, de Santé et de Productions Animales, Département des Sciences et Techniques de Production Animale et Halieutique (DSTPAH), Faculté d’Agronomie (FA), Université de Parakou (UP), Cotonou 01 BP 2115, Benin

**Keywords:** anthelminthic activity, molecular networking, ellagic acid, gallic acid, *Terminalia leiocarpa*, *Haemonchus contortus*, *Caenorhabditis elegans*

## Abstract

*Terminalia leiocarpa* is a medicinal plant widely used in ethnoveterinary medicine to treat digestive parasitosis whose extracts were shown to be active against gastrointestinal nematodes of domestic ruminants. The objective of our study was to identify compounds responsible for this activity. Column fractionation was performed, and the activity of the fractions was assessed *in vitro* on *Haemonchus contortus* and *Caenorhabditis elegans* as well as their cytotoxicity on WI38 fibroblasts. Two fractions were the most active on both nematode models and less cytotoxic. LC-MS/MS analysis and manual dereplication coupled to molecular networking allowed identification of the main compounds: ellagic acid and derivatives, gallic acid, astragalin, rutin, quinic acid, and fructose. Other potentially identified compounds such as shikimic acid, 2,3-(*S*)-hexahydroxydiphenoyl-D-glucose or an isomer, quercetin-3-*O*-(6-*O*-galloyl)-*β*-D-galactopyranoside or an isomer, and a trihydroxylated triterpenoid bearing a sugar as rosamultin are reported in this plant for the first time. Evaluation of the anthelmintic activity of the available major compounds showed that ellagic and gallic acids were the most effective in inhibiting the viability of *C. elegans*. Their quantification in fractions 8 and 9 indicated the presence of about 8.6 and 7.1 µg/mg ellagic acid and about 9.6 and 2.0 µg/mg gallic acid respectively. These concentrations are not sufficient to justify the activity observed. Ellagic acid derivatives and other compounds that were found to be positively correlated with the anthelmintic activity of the fractions may have additive or synergistic effects when combined, but other unidentified compounds could also be implicated in the observed activity.

## 1. Introduction

*Terminalia leiocarpa* (DC.) Baill (Combretaceae) (previously *Anogeissus leiocarpus*) is a 15–18 m tall tree found in India and Africa (especially in West and Central Africa). It is a very important tree because of its high use in traditional medicine, as wood, and in energy production. Indeed, *T. leiocarpa* is widely used by various communities to treat numerous ailments including cough, tuberculosis, diarrhea, dysentery, helminthiasis, malaria, trypanosomiasis, hemorrhoids, skin diseases, fever, and diabetes [1,2,3,4,5]. Several pharmacological studies have concluded that the plant has antibacterial, antioxidant, anthelmintic, and anti-tuberculosis properties [4,6,7].

The anthelmintic activity of *T. leiocarpa* has been already evaluated and the results obtained showed that its leaf extracts were very active on ruminant digestive parasites both *in vitro* and *in vivo*. Indeed, Kabore et al. [8] showed that the aqueous extract of *T. leiocarpa* leaves was very active *in vitro* on eggs, larvae, and adult worms of *H. contortus*, a digestive parasite of small ruminants. The IC_50_ value of the extract was estimated to be 409.5 µg/mL for the inhibition of *H. contortus* egg hatching. Similarly, Ndjonka et al. [6] showed that the ethanol extract of *T. leiocarpa* leaves exhibited strong anthelmintic activity *in vitro* on *C. elegans*. These results were later confirmed by the work of Soro et al. [9] who showed that the ethanol extract of the plant roots was very effective *in vivo* (in sheep) on *H. contortus* and *Trichostrongylus colubriformis* at a concentration of 80 mg/kg orally. Furthermore, a screening of the *in vitro* anthelmintic activity of some of the most common plants used in ethnoveterinary medicine in Benin to treat digestive parasitosis of small ruminants on *H. contortus* larvae migration showed that the MeOH extract of *T. leiocarpa* leaves was one of the most active ones [10].

Although the interesting anthelmintic activity of *T. leiocarpa* has already been established [6,8,9,10], little work has been conducted to identify the molecules responsible for this activity. Ndjonka et al. [11] linked the anthelmintic activity of the plant to phenolic acids including ellagic acid, gentisic acid, and gallic acid. On the other hand, the work of Waterman et al. [2] concluded that punicalagin was partly responsible for the anthelmintic activity of the aqueous extract of the leaves of *T. leiocarpa*. Indeed, the authors considered that the concentration of punicalagin in the extract was too low to justify the strong anthelmintic activity of the plant. There would therefore have to be other anthelmintic compounds that act in addition or synergy with punicalagin. Furthermore, Ademola and Eloff [1] concluded that the anthelmintic activity of *T. leiocarpa* was due to several different compounds, but did not identify them. Thus, in view of the pharmacological importance of this plant in the treatment of digestive parasitosis in ruminants, it appears necessary to identify the main compounds responsible for its anthelmintic activity. This is particularly relevant to identify new anthelmintic molecules in the context of the development of resistance against the synthetic anthelmintics currently used [12,13].

The identification of compounds in an extract is a difficult, tedious, and sometimes time-consuming and expensive task due to the complexity of some plant matrixes. In recent years, molecular networking, an organization and dereplication LC-MS/MS based technique has been developed. This technique allows rapid identification proposals of molecules and their visualization and organization into clusters based on the similarity between their MS/MS fragmentation [14,15,16]. Molecular networking is increasingly used for the tentative identification of natural substances by comparison of the experimental data with reference MS/MS fragmentation spectra [17,18].

In the present study, we combined fractionation of the MeOH extract of *T. leiocarpa* leaves with HPLC-PDA-HRMS/MS analysis and the use of molecular networking to identify the major compounds responsible for its anthelmintic activity. The anthelmintic activity was evaluated individually for identified major compounds commercially available, some of which were quantified by HPLC-PDA.

## 2. Results and Discussion

### 2.1. Cytotoxicity and Anthelminthic Activities of Fractions

We chose the MeOH extract of leaves of *T. leiocarpa* in view of its high anthelmintic activity and low cytotoxicity observed previously [10]. Open column chromatography fractionation of the extract yielded nine fractions labelled 1 to 9. Cytotoxicity of the fractions was evaluated on WI38 cells with the MTT assay. Fractions 1 to 6 showed mild to moderate toxicity with IC_50_ values ranging from 58.9 to 78.9 µg/mL (Table 1). On the other hand, fractions 7, 8, and 9 were considered as not cytotoxic with an IC_50_ greater than 100 µg/mL [18].

The anthelmintic activity of the fractions was evaluated on infective *H. contortus* larvae and young adult of *C. elegans*. These two nematode models are often used to evaluate anthelmintic activity and to identify anthelmintic molecules in natural substances [2,6,8]. Unfortunately, after the cytotoxicity evaluation, the remaining amount of fractions 1 and 3 was not sufficient to evaluate their anthelmintic activity. Nevertheless, these fractions were the two most cytotoxic on WI38 cells, after fraction 6, and would be less interesting to promote as anthelmintic.

On the other hand, fractions 8 and 9 exhibited strong anthelmintic activity (superior to that obtained for the MeOH extract of *T. leiocarpa*) on *H. contortus* and *C. elegans* (Table 1). In addition, fractions 5, 6, and 7 showed moderate anthelmintic activity with inhibition of larval migration ranging from 21.0 to 40.0% at a concentration of 600 µg/mL. The anthelmintic activity of these three fractions on young adult of *C. elegans* was in the same range as that observed on *H. contortus*, with approximately 20% inhibition of viability. These results suggest that anthelmintic compounds are more concentrated in fractions 8 and 9. Furthermore, the lower anthelmintic activity observed in fractions 5, 6, and 7 suggests that these fractions also contain anthelmintic compounds with lower activity or present in lower quantities. These results corroborate the work of Ademola and Eloff [1] who concluded that the anthelmintic activity of *T. leiocarpa* was due to several compounds with various polarities. In general, the anthelmintic activity of the different fractions on *H. contortus* is similar to that observed on *C. elegans*, the two nematodes sharing nearly 70% similarity [19].These results support the use of *C. elegans* in the identification of anthelmintic compounds instead of ruminant parasitic nematodes that are difficult to obtain and maintain in the laboratory [6,11]. Furthermore, the high anthelmintic activity observed in both nematode species and different physiological stages (larva and adult) suggests that the compounds responsible for anthelmintic activity in *T. leiocarpa* could be multitarget. Generally, compounds/extracts with anthelmintic properties show some specificity of action on nematode stages. For example, the water extract of *Daniellia oliveri* leaves was more active on eggs than larvae [8]. Similarly, levamisole is very active on larvae and adult worms but ineffective on eggs [20].

As fractions 8 and 9 were the least toxic and were more active than other fractions, we considered the identification of their major compounds.

### 2.2. Molecular Networking and Major Compounds in the Most Active Fractions

All fractions as well as the MeOH extract, were analyzed by HPLC-PDA-HRMS/MS in negative ion mode. The mass spectrometry data of all fractions (fraction 1 to 9) processed on MZmine 2.5.3 allowed the generation of a spectral alignment with 362 features. The molecular network was built with these 362 features on GNPS and is available on the link: http://gnps.ucsd.edu/ProteoSAFe/status.jsp?task=a9c6688a76d54309817b5a617054b536 (accessed on 10 November 2022).

**Figure 1:**
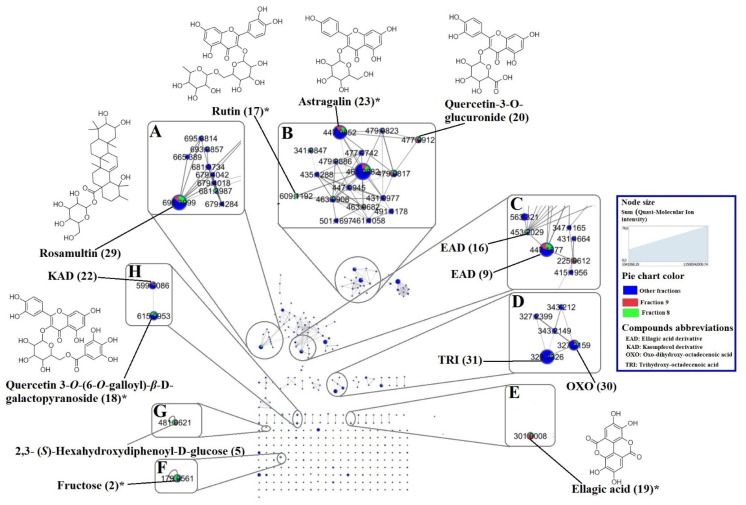
Molecular network of fractions 1 to 9 of *Terminalia leiocarpa* leaves MeOH extract showing the major compounds in fractions 8 (green), 9 (red), and other fractions (blue). The numbers indicate the identification code of the compounds and asterisks show the compounds whose identification was confirmed by injection with the standard. (**A**) triterpenic derivatives, (**B**) *O*-glycosylated flavonoids, (**C**) ellagic acid derivatives, (**D**) fatty acids, (**E**) ellagic acid, (**F**) sugar, (**G**) tannin, (**H**) glycosylated galloylated flavonoids. Edge widths are proportional to the level of similarity (cosine score). The size of the nodes is proportional to the sum of quasi-molecular ion intensity of fractions 1 to 9. * compounds identified by standard comparison.

The constructed molecular network shows the metabolites present in fractions 8 (green nodes) and 9 (red nodes) compared to those observed in fractions 1–7 (blue nodes) (Figure 1). We mainly focused on fractions 8 and 9 because of their stronger anthelmintic activity and lower cytotoxicity. The molecular network classified the detected metabolites into several clusters. In the molecular network presented in Figure 1, we identified triterpenic derivatives (Figure 1A), *O*-glycosylated flavonoids (Figure 1B), ellagic acid derivatives (Figure 1C), fatty acids (Figure 1D), ellagic acid (Figure 1E), sugar (Figure 1F), tannin (Figure 1G), and glycosylated galloylated flavonoids (Figure 1H). This is in accordance with the previously performed phytochemical analyses which showed that *T. leiocarpa* contains mainly triterpenes, phenolic acids including ellagic acid and its derivatives, flavonoids, fatty acids, tannins, and sugars [2,4,7,21].

The dereplication performed on GNPS was completed by manual dereplication comparing HRMS/MS data to existing literature in order to identify the major compounds in fractions 8 and 9. Table 2 presents the mass spectrometry data of the major compounds identified putatively or confirmed with reference standards, in fractions 8 and 9.

**Figure 2:**
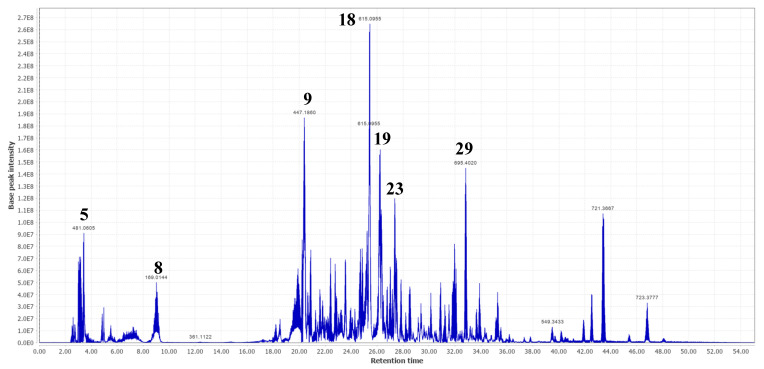
Base peak intensity (BPI) chromatogram of fraction 8 of the MeOH extract of *Terminalia leiocarpa* showing the main compounds identified. (**5**) 2,3-(*S*)-hexahydroxydiphenoyl-D-glucose or isomer; (**8**) gallic acid; (**9**) ellagic acid derivative; (**18**) quercetin-3-*O*-(6-*O*-galloyl)-*β*-D-galactopyranoside or isomer; (**19**) ellagic acid; (**23**) astragalin, and (**29**) rosamultin or isomer.

**Figure 3:**
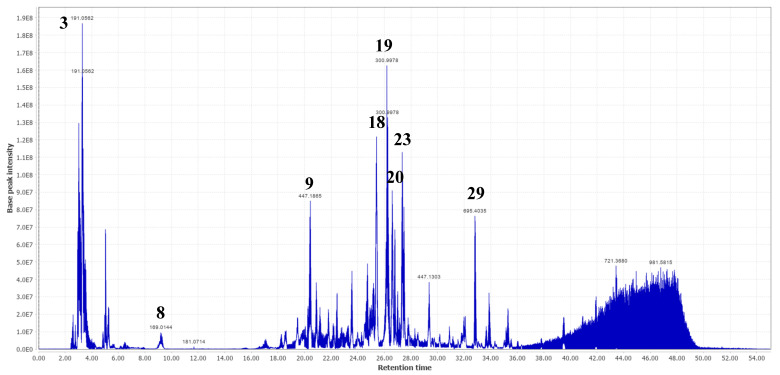
Base peak intensity (BPI) chromatogram of fraction 9 of the MeOH extract of *Terminalia leiocarpa* showing the main compounds identified. (**3**) Quinic acid; (**8**) gallic acid; (**9**) ellagic acid derivative; (**18**) quercetin-3-*O*-(6-*O*-galloyl)-*β*-D-galactopyranoside or isomer; (**19**) ellagic acid; (**20**) quercetin-3-*O*-glucuronide; (**23**) astragalin, and (**29**) rosamultin or isomer.

Compounds (**3**), (**4**), and (**8**) were the first three eluting phenolic acids in fractions 8 and/or 9, which were tentatively identified. Compound (**3**) showed a deprotonated molecular ion [M-H]^-^ at *m*/*z* 191.0562 and one of its MS/MS fragments was observed at *m*/*z* 173.0465 [M-H-H_2_O]^-^. Compound (**8**) showed a signal in the full scan spectrum at *m*/*z* 169.0144 [M-H]^-^ and a main MS/MS fragment at *m*/*z* 125.0247 [M-H-CO_2_]^-^. So, these two phenolic acids were identified as quinic acid (**3**) and gallic acid (**8**). Their identification was confirmed by injection with the corresponding standards. Compounds (**3**) and (**8**) were previously identified in *T. leiocarpa* [2,3]. Compound (**4**) showed a deprotonated [M-H]^-^ ion at *m*/*z* 173.0456 and one of its major MS/MS fragments was observed at *m*/*z* 119.0353. A comparison of the MS/MS data of compound (**4**) with the literature identified it as shikimic acid [22]. To the best of our knowledge, this is the first time the presence of compound (**4**) has been reported in *T. leiocarpa*. Compound (**19**), eluting later, yielded a deprotonated molecular ion [M-H]^-^ at *m*/*z* 300.9978 that fragmented in MS/MS to give two main fragments at *m*/*z* 163.0398 and *m*/*z* 169.0144 (Table 2). Its correspondence to ellagic acid was confirmed by injection of a standard. Like compounds (**3**) and (**8**), ellagic acid (**19**) was previously identified in *T. leiocarpa* extracts [4,7]. It was one of the major compounds in fractions 8 and 9 (Figure 2 and Figure 3, respectively) and can be visualized in the molecular network (Figure 1E).

Compounds (**9**), (**10**), (**11**), (**12**), (**13**), (**14**), (**15**), and (**16**), grouped into cluster 1C (Figure 1) were identified as derivatives of ellagic acid (**19**). Indeed, these compounds showed different molecular ions in the full scan spectrum and a main MS/MS fragment at *m*/*z* 300.9987, corresponding to the ellagic acid fragment (Table 2). This main fragment corresponded to [M-H-146]^-^ for (**9** and **11**), [M-H-176]^-^ for (**10**), [M-H-130]^-^ for (**12** and **15**) and [M-H-152]^-^ for (**14** and **16**), which suggests that the ellagic acid moiety was potentially linked to a deoxyhexoside, a glucuronide, a dideoxyhexoside, or a gallate unit, respectively. However, the Δ ppm obtained for these structural proposals were too high (sometimes >200). Isolation should allow their precise identification and characterization of these compounds will likely identify new compounds in *T. leiocarpa*. The presence of ellagic acid derivatives has long been suspected in some extracts of *T. leiocarpa*, in the genus Terminalia or other Combretaceae species [4,7,23,24,25].

A total of five flavonoids were identified in both fractions. The first eluted at 25.24 min and exhibited a deprotonated molecular ion [M-H]^-^ at *m*/*z* 609.1453 with main MS/MS signals at *m*/*z* 459.1496, *m*/*z* 301.0353 and *m*/*z* 313.0574 (Table 2). A comparison of these MS/MS fragments with those of the literature allowed us to identify compound (**17**) as rutin. Its identification was confirmed by injection of the standard. The compound (**18**) showed a signal in the full scan spectrum at *m*/*z* 615.0955 [M-H]^-^ and two main MS/MS fragments at *m*/*z* 301.0353 and *m*/*z* 313.0553. Since compound (**18**) showed a molar mass close to 2-*O*-galloylhyperin and this compound was available in our laboratory, we injected it to see if it was the same compound. The 2-*O*-galloylhyperin gave the two main MS/MS fragments (*m*/*z* 301.0353 and *m*/*z* 313.0553), like compound (**18**) but had a different retention time. We therefore concluded that these were isomers, and that compound (**18**) could correspond to quercetin-3-*O*-(6-*O*-galloyl)-*β*-D-galactopyranoside or an isomer. The latter was previously identified in *Guiera senegalensis* (Combretaceae) [3] and *Tapirira guianensis* (Anacardiaceae) [26]. To the best of our knowledge, this is the first time that compound (**18**) has been identified in *T. leiocarpa*. Mass spectrometry chromatograms showed that it is also one of the major compounds in fractions 8 and 9 (Figure 2 and Figure 3). Compound (**23**) ([M-H]^-^ at *m*/*z* 447.0932) showed two major MS/MS fragments at *m*/*z* 285.0404 and *m*/*z* 284.0323. It was identified as astragalin [27]. The identification was confirmed by injection of the standard. The compound was previously identified in *Pteleopsis suberosa* (Combretaceae) [27]. To the best of our knowledge, this is the first time this compound has been identified in *T. leiocarpa* where it seems to be present as the major compound of the leaves. The molecular network organized the flavonoids detected into two major groups. Cluster H consists of glycosylated galloylated flavonoids (including compounds **18** and **22**) and cluster B contained *O*-glycosylated flavonoids (including compounds **17**, **20,** and **23**) (Figure 1C,D).

Another major metabolite was eluted at 3.44 min and showed a signal in the full scan spectrum at *m*/*z* 481.0605 [M-H]^-^ with a high MS/MS fragment at *m*/*z* 331.0672. These spectral data are similar to those obtained by Fernandes et al. [28] who identified the compound as 2,3-(*S*)-hexahydroxydiphenoyl-D-glucose, or an isomer (**5**), a hydrolysable tannin. This compound was previously identified in *Terminalia myriocarpa* and *Terminalia calamansanai* [29,30]. To the best of our knowledge, this is the first time that compound (**5**) has been identified in *T. leiocarpa*. Combretaceae in general and species of the genus Terminalia in particular are well known for their high content of hydrolysable tannins [29,30]. Compound (**5**) is one of the major compounds in fraction 8 (Figure 3) and was visualized in the molecular network (Figure 1G).

Compound (**29**) is a trihydroxylated triterpene wearing a sugar, which could correspond to rosamultin, already identified in *Terminalia alata* [31]. Similarly, compounds (**30**) and (**31**) were putatively identified as fatty acids in comparison with literature data [32,33,34]. Compound (**2**) exhibited a deprotonated molecular ion [M-H]^-^ at *m*/*z* 179.0564 and was identified as fructose after injection of the standard, while compound **1** was tentatively identified as an hexitol.

**Table 2:** HPLC-DAD-HRMS/MS data (APCI negative mode) of the major compounds identified in fractions 8 and 9 of the MeOH extract of *Terminalia leiocarpa*.

Code	RT (min)	Molecular Formula	Quasi- Molecular Ion	MS/MS Fragment	Molecular Mass		Error (ppm)	Identification	Isolated Previously from	Source		References
					Observed	Theoretical				F8#	F9#	
**1**	3.02	C_6_H_14_O_6_	181.0715 [M-H]^-^	179.0560 144.0665 101.0245 163.0610	182.0794	182.0790	1.99	Hexitol			√	
**2**	3.17	C_6_H_12_O_6_	179.0564 [M-H]^-^	161.0460 113.0248	180.0643	180.0634	5.06	Fructose *		√	√	
**3**	3.29	C_7_H_12_O_6_	191.0562 [M-H]^-^	181.0715 179.0569 173.0465 189.8369	192.0641	192.0634	3.71	Quinic acid *	*Terminalia ferdinandiana*	√	√	[35]
**4**	3.40	C_7_H_10_O_5_	173.0456 [M-H]^-^	119.0353 129.0198 137.0243 155.0348	174.0535	174.0528	3.89	Shikimic acid	*Anogeissus latifolia*	√	√	[3]
**5**	3.44	C_20_H_18_O_14_	481.0605 [M-H]^-^	331.0672 421.1343 173.0456 300.9998 375.1294	482.0684	482.0697	−2.70	2,3- (*S*)-Hexahydroxydiphenoyl-D-glucose	*T. calamansanai, T. myriocarpa*	√		[29,30]
**6**	4.97	C_9_H_18_O_7_	283.1037 [M+HCOO]^-^	243.0630 273.0739 179.0564	238.1061	238.1053	3.56	n.i		√		
**7**	5.51	C_17_H_26_O_12_	421.1360 [M-H]^-^	375.1310 287.0888 267.0739 357.1195 331.0686	422.1439	422.1424	3.49	n.i		√		
**8**	9.02	C₇H₆O₅	169.0144 [M-H]^-^	125.0247 168.0070 124.0173 126.0283	170.0223	170.0215	4.57	Gallic acid *	*A. leiocarpa, T. ferdinandiana*	√	√	[2,25,35]
**9**	20.43		447.1860 [M-H]^-^	401.1822 300.9978 179.0560				Ellagic acid derivative		√	√	
**10**	20.90		477.1626 [M-H]^-^	431.1540 445.1712 300.9982 169.0147				Ellagic acid derivative		√		
**11**	21.60		447.1515 [M-H]^-^	300.9987 289.0723 387.1662 169.0150				Ellagic acid derivative		√		
**12**	22.43		431.1910 [M-H]^-^	387.1653 169.0145 300.9980 327.1093				Ellagic acid derivative		√	√	
**13**	22.78		387.1660 [M-H]^-^	169.0149 301.0005				Ellagic acid derivative		√		
**14**	23.56		453.1048 [M-H]^-^	387.1666 289.0226 439.0686 169.0143 300.9990 125.0252				Ellagic acid derivative		√	√	
**15**	24.73		431.1912 [M-H]^-^	300.9982 169.0146 289.0718 125.0248 205.1234				Ellagic acid derivative		√	√	
**16**	24.86		453.1979 [M-H]^-^	433.2072 300.9979 407.1930 169.0145				Ellagic acid derivative		√		
**17**	25.24	C_27_H_30_O_16_	609.1453 [M-H]^-^	459.1496 301.0353 313.0574 567.2086 169.0144	610.1532	610.1534	−0.30	Rutin *	*A. leiocarpa*	√		[21]
**18**	25.44	C_28_H_24_O_16_	615.0955 [M-H]^-^	301.0353 313.0553 565.2844 463.0887 169.0144	616.1034	616.1064	−4.93	Quercetin-3-*O*-(6-*O*-galloyl)-*β*-D-galactopyranoside	*T. guianensis*	√	√	[26]
**19**	26.22	C_14_H_6_O_8_	300.9978 [M-H]^-^	163.0398 169.0144	302.0057	302.0063	−1.88	Ellagic acid *	*A. leiocarpa Terminalia brownii*	√	√	[4,7]
**20**	26.59	C_21_H_18_O_13_	477.0677 [M-H]^-^	301.0354 302.0383 169.0145 439.0670 151.0035 289.0715 287.0564	478.0756	478.0747	1.80	Quercetin-3-*O*-glucuronide			√	
**21**	26.79	C_48_H_68_O_5_	723.5013 [M-H]^-^	439.0679 169.0140 463.0896 303.0508 289.0721 677.5002 125.0249	724.5092	724.5067	3.48	n.i			√	
**22**	27.02	C_28_H_24_O_15_	599.1047 [M-H]^-^	435.1282 285.0406 473.1672 313.0556	600.1126	600.1115	1.80	Kaempferol linked to gallate and deoxy-hexose		√	√	
**23**	27.35	C_21_H_20_O_11_	447.0932 [M-H]^-^	285.0404 284.0323 439.0670 442.7359 289.0715	448.1011	448.1006	1.20	Astragalin *	*P. suberosa*	√	√	[27]
**24**	27.85	C_37_H_60_O_14_	727.3909 [M-H]^-^	565.3358 519.3334 439.0675 477.1035 343.2121	728.3988	728.3983	0.68	n.i		√		
**25**	28.53	C_37_H_60_O_13_	711.3926 [M-H]^-^	343.2126 371.1710 531.1526 439.0681 583.1072 289.0722	712.4005	712.4034	−4.06	n.i		√		
**26**	29.36	C_22_H_24_O_10_	447.1303 [M-H]^-^	439.0685 303.0513 169.0151 287.0574 289.0725 125.0251	448.1382	448.1369	2.80	n.i			√	
**27**	30.90	C_30_H_62_O_18_	709.3831 [M-H]^-^	507.2063 547.3296 501.3242 597.1829 461.1088	710.3910	710.3936	−3.68	n.i		√	√	
**28**	31.96	C_37_H_60_O_13_	711.3934 [M-H]^-^	549.3431 697.3820 503.3406 695.4033 702.6718	712.4013	712.4034	−2.94	n.i		√		
**29**	32.82	C_36_H_58_O_10_	695.4020 [M + HCOO]^-^	487.3446 173.9751 686.9651 533.3465	650.4043	650.4030	2.00	Rosamultin or isomer	*T. alata*	√	√	[31]
**30**	33.88	C_18_H_32_O_5_	327.2178 [M-H]^-^	324.4868 211.1343 289.0727 171.1030 229.1447	328.2257	328.2250	2.21	Oxo-dihydroxy-octadecenoic acid	*Globularia* spp. *Bituminaria bituminosa* *Sasa veitchii*	√	√	[32,33,34]
**31**	35.28	C_18_H_33_O_5_	329.2329 [M-H]^-^	211.1343 116.0257 229.1447 326.4767 169.0144	330.2408	330.2406	0.53	Trihydroxy-octadecenoic acid	*Globularia* spp. *B. bituminosa* *S. veitchii*	√	√	[32,33,34]
**32**	43.40	C_34_H_58_O_16_	721.3667 [M-H]^-^	675.3602 397.1340 712.4544 277.2173	722.3746	722.3725	2.93	n.i		√	√	
**33**	46.81	C_34_H_60_O_16_	723.3777 [M-H]^-^	677.3750 116.0257 397.1347 167.0363 119.0260	724.3856	724.3881	−3.50	n.i		√		

RT Retention time; n.i non-identified; * compounds identified by standard comparison; F8 fraction 8; F9 fraction 9, # detected by MS ion ca.

### 2.3. Prediction of Anthelmintic Activity of Features Detected in T. leiocarpa Fractions by Pearson Correlation

**Figure 4:**
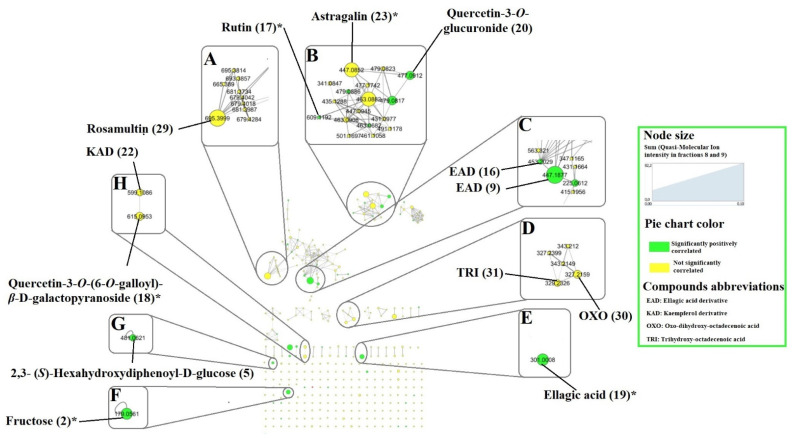
Molecular network of fractions of MeOH extract of *Terminalia leiocarpa* leaves showing compounds significantly positively (green nodes) or not significantly (yellow nodes) correlated with the anthelmintic activity on *C. elegans*. The numbers indicate the identification code of the compounds and asterisks show the compounds whose identification was confirmed by the injection of a standard. (**A**) triterpenic derivatives, (**B**) *O*-glycosylated flavonoids, (**C**) ellagic acid derivatives, (**D**) fatty acids, (**E**) ellagic acid, (**F**) sugar, (**G**) tannin, (**H**) glycosylated galloylated flavonoids. Edge widths are proportional to the level of similarity (cosine score). The size of the nodes is proportional to the sum of quasi-molecular ion intensity of fractions 8 and 9. * compounds identified by standard comparison.

Phytochemical analysis of the fractions of *T. leiocarpa* showed that they contain a variety of metabolites. Thus, in order to identify those potentially responsible for anthelmintic activity, we calculated the correlation coefficient between the intensity of the different metabolites within each fraction and the anthelmintic activity of the fractions on *C. elegans*. To achieve this, we added the anthelmintic activity on *C. elegans* of the nine fractions to the spectral alignment file containing the 362 features detected. The file was then exported to RStudio to calculate the Pearson correlation coefficient between the detected features and the anthelmintic activity. The calculated correlation coefficient along with the probability (*p*-value) was used to generate a three-value score (−1, 0 and +1) which was then imported into Cytoscape to visualize the features/compounds. A −1 signifies a significant (*p* < 0.05) negative correlation between the feature and anthelmintic activity on *C. elegans*. In other words, these compounds would exhibit antagonistic activity. On the other hand, a +1 means a significant (*p* < 0.05) positive correlation between the feature concerned and the anthelmintic activity on *C. elegans*. Compounds represented in the molecular network by green colored nodes are significantly positively correlated to the anthelmintic activity (Figure 4). This means that these compounds could have anthelmintic activity and could partially account for the anthelmintic activity of the fractions on *C. elegans*. Those not significantly (*p* > 0.05) correlated with the anthelmintic activity of the fractions on *C. elegans* are represented in yellow.

The results showed that 43 features (11.88%) were significantly positively correlated with anthelmintic activity compared to 318 (87.85%) that were not significantly correlated, and only one feature (0.27%) was significantly and negatively correlated with anthelmintic activity. The results showed that ellagic acid (**19**) and its derivatives (compound **9** for example) were more abundant in fractions 8 and 9, and positively correlated with the anthelmintic activity of the fractions (Figure 4C,E). They may explain at least in part the higher activity of fractions 8 and 9 as several of them are present in higher quantities in these fractions. Ellagic acid (**19**) is known to possess interesting anthelmintic activity on *H. contortus* and *C. elegans* [11,36]. Like ellagic acid (**19**), some flavonoids identified in fractions 8 and 9 were positively correlated with anthelmintic activity on *C. elegans* (Figure 4B,D). These include compounds (**17**) and (**20**). Many studies have shown that flavonoids are endowed with anthelmintic activity on various nematodes [37,38,39]. Nevertheless, all flavonoids are not correlated with anthelminthic activities, as we observed that astragalin (**23**) which was quite abundant in the most active fractions did not possess a significant correlation with anthelmintic activity (Figure 4B). The same was observed for the triterpenic derivative tentatively identified as rosamultin (**29**) (Figure 4A). A significant positive correlation was also observed for compound (**5**) (a hydrolysable tannin). Previously conducted studies show that tannins are endowed with strong anthelmintic activity [38], but this activity may depend on the type and structure of the tannins that are present. A positive correlation was also observed for fructose (**2**), which is a common sugar that should not have anthelminthic activity, but whose polarity may be close to active compounds.

The results of the correlation between the detected metabolites and the anthelmintic activity on *C. elegans* of the fractions remain indicative and should be taken with caution. Indeed, antagonistic, additive, or synergistic activity are possible when compounds are in a mixture, and their activity in these extracts is not related to their activity when tested individually. These results are nevertheless a lead towards an identification of anthelmintic molecules in *T. leiocarpa*. The evaluation of the anthelmintic activity of each compound and several mixtures would allow the confirmation or not of the results of the correlation.

### 2.4. Anthelmintic Activity of Major Compounds and Their Quantification

**Figure 5:**
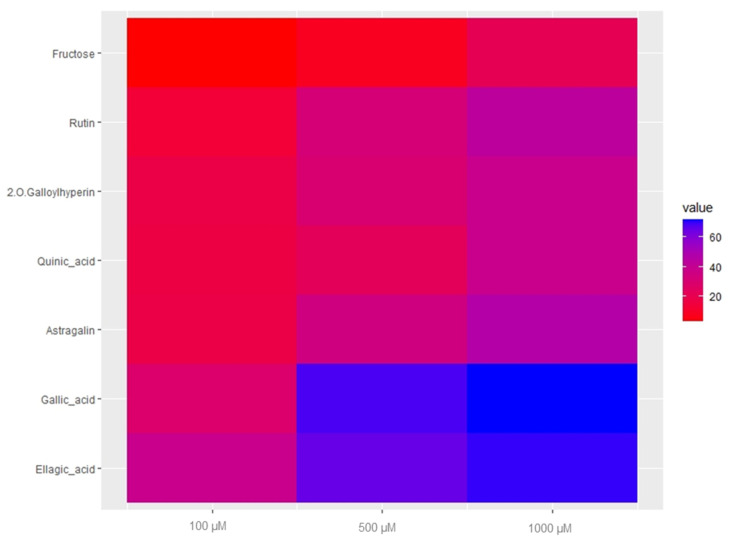
Heatmap of viability inhibition rate (%) (minus that of the negative control) of young *Caenorhabditis elegans* adults treated with the compounds at different concentrations.

The anthelmintic activity of the major identified and commercially available compounds of fractions 8 and 9 was evaluated in order to confirm or not the predictions in Section 2.3 and to determine if these compounds could be responsible for the anthelmintic activity of the MeOH extract of *T. leiocarpa*. The results of the anthelmintic activity of available standards are presented in Figure 5.

In general, the different compounds inhibited the viability of young adults of *C. elegans*. The anthelmintic activity varied with the compounds and concentrations tested. Ellagic acid (**19**) and gallic acid (**8**) were the most effective with a reduction in viability approaching 70% at 500 µM (compound **19**: 151.1 µg/mL and compound **8**: 85.06 µg/mL). These results confirm the positive correlation between the anthelmintic activity of the fractions and these compounds. Furthermore, studies conducted previously had concluded that compounds (**8**) and (**19**) have strong anthelmintic activity on *H. contortus* and *C. elegans* [11,36]. Astragalin (**23**) and rutin (**17**) moderately inhibited the viability of *C. elegans* at the highest concentration tested (1000 µM) (compound **17**: 610.5 µg/mL and compound **23**: 448.4 µg/mL) and the inhibition rate for both compounds was around 50% (Figure 5). The moderate anthelmintic activity of compound (**23**) confirms the absence of significant correlation between the anthelmintic activity of the fractions and this feature (Figure 4D). The anthelmintic activity of compound (**23**) was previously evaluated on *Fasciolopsis buski*, a parasitic trematode of pig [40]. To our knowledge, this is the first time that the anthelmintic activity of astragalin has been evaluated on *C. elegans*. Rutin (**17**) on the other hand showed low anthelmintic activity despite its strong anthelmintic activity prediction. Previous work also showed weak anthelmintic activity of compound (**17**) on *H. contortus* [37,38]. Like the other flavonoids, 2-*O*-galloylhyperin (an isomer of compound **18**) moderately inhibited the viability of adult *C. elegans* worms at a concentration of 1000 µM (616.5 µg/mL). This activity seems low in view of the significant positive correlation between compound (**18**) (its isomer) and the anthelmintic activity of the fractions, but as the structures are different, we cannot draw a conclusion.

Furthermore, the major available identified compounds that showed highest anthelmintic activity were quantified in the most active fractions (fractions 8 and 9). Astragalin (**23**) which showed moderate anthelmintic activity was also quantified in the two most active fractions, as it could serve as an analytical marker, given its high concentration. As some ellagic acid derivatives were significantly positively correlated with anthelmintic activity (Figure 4A), we also quantified the ellagic acid derivatives in ellagic acid equivalents. The results are presented in Table 3 and Table 4. The concentrations of ellagic acid (**19**) and gallic acid (**8**) in fraction 8 were estimated to be 8.6 ± 0.7 and 9.7 ± 0.8 µg/mg of the fraction (Table 3). These compounds were most concentrated in fraction 8 compared to astragalin (**23**) which accounted for only 0.8 ± 0.1 µg/mg of this fraction. Compounds (**8**) and (**19**) were more concentrated in fraction 8 while compound (**23**) was more concentrated in fraction 9 (Table 3). The total concentrations of ellagic acid derivatives (**9**–**16**) were 2.1 and 6.0 µg of ellagic acid equivalents/mg fraction respectively in fractions 8 and 9 (Table 4). The concentration of ellagic acid derivatives plus ellagic acid in fraction 8 (10.8 µg/mg of fraction) was lower than that obtained in fraction 9 (13.2 µg/mg of fraction).

## 3. Materials and Methods

### 3.1. Chemicals and Reagents

HPLC grades of hexane, dichloromethane (DCM), and methanol (MeOH) were purchased from VWR International (Radnor, PA, USA). WI38 cells (non-cancerous human fibroblast cell line) were obtained from LGC standards (Molsheim, France). Dimethylsulfoxide (DMSO), camptothecin, (3-(4,5-dimethylthiazol-2-yl)-2,5-diphenyltetrazolium bromide (MTT)), levamisole, and ellagic acid (**19**) were purchased from Sigma-Aldrich (Bornem, Belgium). Penicillin and streptomycin were purchased from Lonza (Verviers, Belgium). 2-*O*-galloylhyperin was purchased from MedChemTronica (Sollentuna Sweden). Astragalin (**23**) was purchased from AmBeed (Arlington, USA). Gallic acid (**8**) and rutin (**17**) were purchased from Sigma-Aldrich (Steinheim, Germany). Quinic acid (**3**) was purchased from Tokyo Chemical Industry (TIC) Europe NV (Zwijndrecht, Belgium). Fructose (**2**) was purchased from Merck (Darmstadt, Germany).

### 3.2. Plant Collection

Fresh leaves of *T. leiocarpa* were collected in North Benin (N’Dali municipality). The sample was authenticated at the National Herbarium of Benin (NHB), University of Abomey-Calavi, Benin (AAC 1504/HNB). The leaves were washed with water to remove dust and other contaminants before being dried in the laboratory at 25 °C for 2 weeks. The dry leaves were ground in a 0.5 mm diameter mill. The powder obtained was stored in hermetically sealed boxes at 25 °C in the laboratory.

### 3.3. Extraction Procedure

The extraction procedure has been described previously [10]. Briefly, 250 mL of hexane was added to 50 g of powder and the mixture was macerated on a shaker for 12 h. After filtration, a second 250 mL portion of hexane was used for a further 12 h maceration under shaking. The same procedure was repeated for dichloromethane (DCM) and MeOH on the same powder sample. The MeOH extract was evaporated with a rotavapor, weighed, transferred to labelled boxes, and stored at +4 °C.

### 3.4. Open Column Chromatography (OCC) Fractionation of T. leiocarpa MeOH Extract

A series with thin layer chromatography (TLC) was performed to identify the solvent system to be used for the fractionation of the MeOH extract of *T. leiocarpa*. Twenty grams of extract was solubilized in MeOH and added to 50 g of silica gel (0.063–0.2 mm), mixed, and evaporated. In parallel, a silica column was mounted (350 g silica gel in DCM in a glass column: 33 × 35 cm). Extract mixed with silica gel was deposited on the top of the mounted column and the different solvent systems were prepared to elute the column (Table 5). TLC (on silica gel and using the solvent system used to elute the column as mobile phase) was performed in parallel to pool the collected fractions. The plates were sprayed with sulfuric anisaldehyde solution [41] and heated. Sub-fractions were formed by mixing the fractions showing a similar TLC profile. They were dried and stored at +4 °C until use.

### 3.5. Fractions Cytotoxicity

Cytotoxicity of the fractions was evaluated on WI38 cells (non-cancerous human fibroblast cell line) using MTT-assay according to a procedure described in the literature [42]. They were solubilized in DMSO at a concentration of 20 mg/mL. Then 5000 cells per well were seeded overnight in 96-well plates in 180 µL of DMEM (Dulbecco’s Modified Eagle’s Medium supplemented with 10% inactivated fetal calf serum and 1% penicillin/streptomycin). Solubilized extract/fractions (20 mg/mL) were diluted with DMEM to give concentrations from 0.5 to 1000 µg/mL and 20 µL of each diluted solution was added to the seeded cells in each well. Final concentrations tested ranged from 0.05 to 100 µg/mL. After 72 h of incubation, medium was replaced by 100 µL of MTT ([3-(4,5-dimethylthiazol-2-yl)-2,5-diphenyl tetrazolium bromide] tetrazolium salt) solution to measure the metabolic activity of the cells, an indicator of cell viability. MTT solution was prepared by dissolving 15 mg of MTT in 50 mL (5 mL of PBS and 45 mL of DMEM). After 45 min, MTT solution was replaced with an equal volume of DMSO, and absorbance was measured with a spectrophotometer (SpectraMax M3) at 570 and 620 nm to measure formazan formed by the reduction of MTT. The assay was repeated twice in duplicate.

### 3.6. Anthelmintic Activities of Fractions

#### 3.6.1. Viability of *C. elegans* Adult Worms Treated with Fractions and Pure Compounds

Anthelmintic activity of the fractions was evaluated on young adults of the wild type (N2) strain of *C. elegans*. The young adults were provided by the Laboratory of Neurophysiology (Neuroscience Institute, Université Libre de Bruxelles, Brussels, Belgium). Briefly, ten young *C. elegans* adults were manually transferred into each well of a 48-well plate containing 250 µL of M9 buffer solution. Two hundred and fifty microliters of each sample (at a concentration of 1200 µg/mL in M9 buffer) was added to the worms. The final concentration tested for each fraction was 600 µg/mL. This dose was shown to be very discriminating in recently published work [10] and could enable us to easily distinguish the most active fractions from the less active ones. Viability of young adults of *C. elegans* was measured under a binocular microscope after 24 h incubation. Worms that were elongated and immobile even after shaking were considered dead or non-viable [11]. Levamisole was used as a positive control at a concentration of 25 µM. Each treatment was tested in duplicate, and the assay was repeated twice.

Anthelmintic activity of the pure compounds was also evaluated on young adults of *C. elegans*. The assay was conducted in the same way as for the fractions but at three concentrations: 100, 500, and 1000 µM. Each treatment was tested in triplicate and the assay was repeated twice.

#### 3.6.2. Larval Migration Inhibition Assay (LAMIA)

Anthelmintic activity of the fractions was also evaluated on infested larvae (L3) of *H. contortus* using the larval migration inhibition assay (LAMIA). The larvae were obtained by artificially infesting sheep with a pure strain of *H. contortus* provided by the Laboratoire d’Ethnopharmacologie et de Santé Animale (LESA), University of Abomey-Calavi. The droppings of the infested sheep were cultured in the laboratory for ten days and the larvae were collected by the Baermann device. The collected larvae were stored at +4 °C for three months before use. The methodology used for LAMIA was described in the literature [10,43]. In summary, larvae (1000 L3s/mL) were incubated at 25 °C with the fractions at a concentration of 600 µg/mL in phosphate-buffered saline (PBS) solution. After three hours of incubation, the larvae were washed by centrifugation (67× *g*) with PBS solution and deposited on inserts (20 µm diameter) for migration for 3 h. The inserts were previously deposited on canonical tubes, containing PBS solution. After 3 h, the larvae contained in the inserts were discarded and those that migrated into the canonical tubes under the inserts were recovered and counted under the microscope. The rate of inhibition of larval migration was calculated according to the following formula:

$$A=\frac{T-M}{T}\times 100$$

where A is the rate of inhibition of larval migration, T is the total number of larvae deposited on the insert, and M is the number of larvae counted in the canonical tube.

### 3.7. HPLC-PDA-HRMS/MS Analysis

*T. leiocarpa* fractions as well as MeOH extract were analyzed by HPLC-PDA-HRMS/MS to identify major compounds. HPLC-PDA (Thermo Scientific Accela LC Systems) coupled with mass spectrometry (Thermo Scientific LTQ orbitrap XL, Bremen, Germany) constituted the system used for analysis. Instruments were controlled using Thermo Scientific Xcalibur X software. HPLC separation was performed on a Luna C18 column, 250 × 4.6 mm packed with 5 µm particles. The mobile phase consisted of water + 0.1% formic acid (A) and 100% acetonitrile (B). The gradient used for elution was as follows: 0–10 min, 95% A; 10–40 min, 95–40% A; 40–45 min, 40% A; 45–50 min, 40–95% A, and 50–55 min, 95% A. Samples to be analyzed (10 mg/mL) were solubilized in MeOH and 20 µL was injected per fraction. Standards were prepared at a concentration of 500 µg/mL and 20 µL was injected. HRMS/MS analyses were performed in APCI (atmospheric pressure chemical ionization) in positive and negative modes with the following input conditions for the negative mode: capillary temperature 250 °C; APCI vaporizer temperature 400 °C; sheath gas flow rate 20 a.u.; auxiliary gas flow rate 5 a.u. and sweep gas flow rate 5 a.u. For positive mode: capillary temperature 250 °C; APCI vaporizer temperature 400 °C; sheath gas flow 25 a.u.; auxiliary gas flow 25 a.u. and sweep gas flow 5 a.u.; discharge current of 5 µA; capillary voltage of 21 V, and tube lens voltage of 75 V. Chromatograms were recorded between 200 and 600 nm.

### 3.8. Data Processing on MZmine

Raw mass spectrometry data of the fractions as well as that of MeOH extract of *T. leiocarpa* were pre-processed in the MZmine software (version 2.5.3). We only worked with the negative data as they were more sensitive and more informative in comparison with the positive mode. In summary, an ion list was generated by setting the noise level to 1.5 × 10^5^ and 1, respectively for MS1 and MS2. The ion list thus created was used to construct the chromatogram with the MZmine ADAP Chromatogram builder function. The minimum number of scans in the cluster was set to 5. The group intensity threshold and the highest minimum intensity were set to 1.5 × 10^5^. Deconvolution of the constituted ion list was performed using the wavelets (ADAP) algorithm. Deconvolution was performed by setting the main parameters as follows: S/N threshold: 6, SN estimator: intensity window S/N, minimum feature height: 120.000, coefficient/area threshold: 3, peak duration: 0.00 to 0.50, and RT wavelet range: 0.00 to 0.10. Isotopes were grouped using the “Isotope grouper” function and setting the *m*/*z* tolerance to 10 ppm and the RT tolerance to 0.3 min (absolute). Lists of deisotoped ions were aligned by setting the parameters at the same level as for isotope grouping (*m*/*z* tolerance: 10 ppm and RT tolerance: 0.3 min absolute). The list of aligned ions was filtered by removing duplicate peaks (*m*/*z* tolerance: 0.02 and RT tolerance: 0.4 min absolute) and using the “Feature list rows filter” function. The aligned list was deisotoped, gap-filled, and exported as a .csv and .mgf file for submission to GNPS-FBMN (Global Natural Product Social Molecular Networking-Feature Based Molecular Networking).

### 3.9. Dereplication on GNPS

Exported MZmine files as well as the raw mass spectrometry data of the fractions were sent to the GNPS platform using Win SCP software (version 5.21.2). The *m*/*z* tolerance for MS1 and MS2 was set to 0.02 Da by default. Molecular networking was created on the GNPS platform (http://gnps.ucsd.edu (accessed on 10 November 2022)), version 28.2. Nodes were filtered to have a cosine score greater than 0.7 and at least 6 matched peaks. Dereplication against GNPS libraries was set to a cosine score of 0.7, with at least 6 matching peaks. The molecular network was finally processed and visualized on Cytoscape (version 3.8.2).

### 3.10. Quantification of Major Compounds

The quantification of the major compounds from fractions 8 and 9 was performed using an HPLC-PDA system (Accela Thermo ScientificTM, Bremen, Germany) based on the UV absorbance of the compounds. The system was controlled using ChromQuest software (version 4.2.34). Separation of compounds was performed on a Luna C18 (250 × 4.6 mm, 5 μm particles). The mobile phase consisted of water + 0.1% formic acid (A) and 100% acetonitrile (B). The column was eluted in gradient mode: 0–10 min, 5–12% B; 10–20 min, 12–18% B; 20–45 min, 18–25% B; 45–46 min, 25–5% B; and 46–55 min, 5% B. This gradient is different from that used for LC-MS/MS analysis and was intended to allow for better separation of compounds to facilitate quantification. The quantification was performed with an injection of 20 µL and a flow rate of 700 µL/min. The standard compounds to be quantified were prepared at different concentrations in MeOH (HPLC grade) varying from 150 to 25 µg/mL for compounds (**8**) and (**19**) and 50 to 5 µg/mL for compound (**23**). Samples of fractions 8 and 9 were solubilized in MeOH (HPLC grade) at a concentration of 10 mg/mL. The PDA wavelength was set between 220 and 360 nm and the chromatogram was integrated at 254 nm. The assay was conducted in triplicate and repeated three times. The limit of detection (LOD) and limit of quantification (LOQ) were determined from the residual standard deviation (σ) of the regression curves and slopes (S), according to the following equations: LOD = 3.3 σ/S and LOQ = 10 σ/S [18].

### 3.11. Statistical Analysis

Means ± standard deviation of the rate of inhibition of larval migration (*H. contortus*) and viability of young adult worms (*C. elegans*) were calculated for each fraction and control tested. The Pearson correlation coefficient between the metabolite intensities and anthelmintic activity of the fractions and the MeOH extract of *T. leiocarpa* was determined by the methodology developed by Nothias et al. [44]. Analysis was performed on RStudio software (Version 1.4.1717). The calculated correlation coefficient was used to identify on the molecular network, the metabolites whose intensities are significantly correlated (*p* < 0.05) or not, with the anthelmintic activity of the fractions on *C. elegans*.

## 4. Conclusions

In summary, our study identified several compounds in the most active fractions on adult *C. elegans* worms. Several of these compounds had already been previously identified in *T. leiocarpa*. These include quinic acid (**3**), gallic acid (**8**), ellagic acid (**19**), and rutin (**17**). On the other hand, shikimic acid (**4**), 2,3-(*S*)-hexahydroxydiphenoyl-D-glucose (**5**), or an isomer, quercetin-3-*O*-(6-*O*-galloyl)-*β*-D-galactopyranoside (**18**) or an isomer, and a glycosylated trihydroxylated triterpene, as rosamultin (**29**) were identified for the first time in the plant as well as several ellagic acid derivatives. The results of the biological activity prediction showed that several of these compounds are significantly positively correlated with the anthelmintic activity of the fractions on *C. elegans*. Evaluation of the anthelmintic activity of the major available compounds identified showed that gallic acid (**8**) and ellagic acid (**19**) were the most active. The other compounds tested moderately inhibited the viability of *C. elegans*. These results suggest an additive/synergistic effect of the different compounds present but may also indicate that some active substances may not have been identified by our LC-MS method. Further studies could focus on the verification of this hypothesis as well as the isolation and characterization of the other compounds positively and significantly correlated with the anthelmintic activity of the fractions. The anthelmintic activity of these compounds could be evaluated as well as their mechanism of action.

## Figures and Tables

**Table 1:** Migration inhibition rate (%) of *Haemonchus contortus* L3 larvae, mortality rate (%) of *Caenorhabditis elegans* young adult worms treated with *Terminalia leiocarpa* MeOH extract and its fractions at a concentration of 600 µg/mL and cytotoxicity (IC_50_) on fibroblast cells (WI38).

Sample/Control	*H. contortus* (%)	*C. elegans* (%)	Cytotoxicity, IC_50_ (µg/mL)
MeOH	63.4 ± 0.8	70.0 ± 7.1	>100
1	-	-	64.5 ± 5.7
2	29.8 ± 17.5	2.5 ± 4.3	58.9 ± 2.9
3	-	-	64.1 ± 2.2
4	15.1 ± 6.2	12.5 ± 4.3	60.3 ± 10.0
5	21.0 ± 3.9	22.5 ± 4.3	59.6 ± 4.9
6	35.7 ± 11.7	20.0 ± 7.1	78.9 ± 2.0
7	39.9 ± 7.9	22.5 ± 4.3	>100
8	69.4 ± 10.4	75.0 ± 5.0	>100
9	76.4 ± 1.8	85.0 ± 5.0	>100
DMSO (0.5%)	3.1 ± 0.8	0	-
LEV (25µM)	100.0 ± 0.0	100.0 ± 0.0	-

I MeOH: methanol extract of *T. leiocarpa* 1: fraction 1, … 9: fraction 9, DMSO: dimethylsulfoxide, LEV: levamisole, -: not tested.

**Table 3:** Concentration of the major compounds in the two most active fractions.

Compound (ID)	Equation	R^2^	LOD (µg/mL)	LOQ (µg/mL)	F8		F9	
					µg/mL	µg/mg of Fraction	µg/mL	µg/mg of Fraction
Ellagic acid (**19**)	y = 228815x + 926041	0.999	5.5	16.5	86.4 ± 6.9	8.6 ± 0.7	71.4 ± 2.6	7.1 ± 0.3
Astragalin (**23**)	y = 45696x + 15964	0.997	3.3	9.9	7.7 ± 1.2	0.8 ± 0.1	9.6 ± 0.4	1.0 ± 0.0
Gallic acid (**8**)	y = 8197.3x + 26875	0.995	10.3	31.1	96.5 ± 7.8	9.7 ± 0.8	19.9 ± 0.5	2.0 ± 0.5

LOD Limit of Detection, LOQ Limit of Quantification, ID codes, F8 Fraction 8, F9 Fraction 9.

**Table 4:** Concentration of ellagic acid derivatives (in ellagic acid equivalents) in the two most active fractions.

Compound (ID)	F8		F9	
	µg/mL	µg/mg of Fraction	µg/mL	µg/mg of Fraction
Ellagic derivative (**9**)	1.0 ± 0.4	0.1 ± 0.0	13.6 ± 1.1	1.4 ± 0.1
Ellagic derivative (**10**)	2.1 ± 0.3	0.2 ± 0.0	-	-
Ellagic derivative (**11**)	4.2 ± 2.0	0.4 ± 0.2	35.9 ± 3.6	3.6 ± 0.4
Ellagic derivative (**12**)	-	-	-	-
Ellagic derivative (**13**)	2.4 ± 0.8	0.2 ± 0.1	0.5 ± 1.4	0.1 ± 0.2
Ellagic derivative (**14**)	-	-	8.0 ± 0.1	0.8 ± 0.0
Ellagic derivative (**15**)	0.3 ± 0.5	0.1 ± 0.1	0.4 ± 1.0	<LOQ
Ellagic derivative (**16**)	11.1 ± 0.6	1.1 ± 0.1	1.8 ± 0.2	0.2 ± 0.0

ID codes, F8 Fraction 8, F9 Fraction 9.

**Table 5:** Solvent system used for the fractionation of the MeOH extract of *Terminalia leiocarpa*.

Solvent System	Ratio	Volume (mL)
DCM-MeOH	100–0	300
DCM-MeOH	99.5–0.5	400
DCM-MeOH	99–1	600
DCM-MeOH	98–2	600
DCM-MeOH	96–4	600
DCM-MeOH	94–6	1000
DCM-MeOH	92–8	600
DCM-MeOH	90–10	1000
DCM-MeOH	85–15	1200
DCM-MeOH	80–20	1200
DCM-MeOH	70–30	1200
DCM-MeOH	60–40	1200

DCM: Dichloromethane MeOH: Methanol.

## Data Availability

All data generated or analyzed during this study are included in this manuscript.
